# Adrenal insufficiency after curative-intent gastric cancer treatment: a case report

**DOI:** 10.1186/s13256-023-03858-5

**Published:** 2023-04-11

**Authors:** Johan Hardvik Åkerström, Cecilia Radkiewicz

**Affiliations:** 1 Upper Gastrointestinal Cancer Unit, Karolinska University Hospital, Stockholm, Sweden; 2 Upper Gastrointestinal Surgery, Department of Molecular Medicine and Surgery, Karolinska Institutet, Retzius Väg 13 a, Level 4, 171 77 Stockholm, Sweden

**Keywords:** Stomach neoplasms, Adrenal insufficiency, Chemotherapy, Radiosurgery, Case report

## Abstract

**Background:**

Adrenal insufficiency is a life-threatening condition, and advanced gastric cancer is associated with very poor prognosis. Adrenalectomy and/or metastatic invasion of the adrenal glands can cause primary adrenal insufficiency, which in turn can present with symptoms mimicking advanced cancer.

**Case presentation:**

Herein we report of a 68-year-old White male with a history of left adrenalectomy in conjunction with distal gastrectomy due to gastric adenocarcinoma. At the 2-year follow-up visit after cancer surgery, the patient presented with fatigue, unintentional weight loss, hyperkalemia, and a computed tomography scan with a right adrenal mass. Primary adrenal insufficiency caused by gastric cancer metastatic invasion of the remaining right adrenal gland was established and glucocorticoid therapy initiated. The patient received first line palliative chemotherapy with systemic disease control and subsequent stereotactic body radiotherapy to the right adrenal gland. More than 17 months after pathology-confirmed gastric cancer relapse, there is no clinical nor radiological evidence of active malignant disease and the patient is doing well on glucocorticoid replacement therapy.

**Conclusions:**

This case does not only illustrate the importance of prompt and correct clinical management of adrenal insufficiency, but also that selected patients with advanced gastric cancer can gain from and achieve long-term survival using a multimodal treatment approach.

**Supplementary Information:**

The online version contains supplementary material available at 10.1186/s13256-023-03858-5.

## Background

Despite declining incidence over the past 50 years, gastric adenocarcinoma still constitutes a major global health burden and is currently the fifth most common malignancy worldwide [1, 2]. Early-stage gastric adenocarcinoma is often curable [2]. Unfortunately, more than 40% of patients present with advanced gastric cancer, which entails very poor prognosis and a median overall survival of less than a year among those fit for chemotherapy [1–3]. The most frequent sites of metastases are the liver and peritoneum, while the adrenal glands are less commonly invaded [4]. Immediate impact on health is, however, potentially larger due to the risk of adrenal insufficiency, which can present acutely as a life-threatening adrenal crisis. Adrenal insufficiency is the clinical manifestation of deficient production or action of glucocorticoids. The underlying causes to adrenal failure can be primary or central (secondary or tertiary) [5]. Addison’s disease (that is, autoimmune adrenalitis) is the most common cause of primary adrenal insufficiency in developed countries, while long-term administration of exogenous glucocorticoids and prolonged suppression of hypothalamic secretion of corticotropin-releasing hormone is the most frequent risk factor for central adrenal insufficiency [5, 6]. Adrenal metastases are not rare and glucocorticoid therapy is very common in patients with advanced cancer, stressing the importance of an awareness of the mechanisms of adrenal insufficiency among all health professionals involved in clinical cancer care.

## Case presentation

A 68-year-old White male presented with fatigue and unintentional weight loss at the 2-year follow-up visit after distal gastrectomy and lymphadenectomy due to locally advanced gastric adenocarcinoma. The medical history included hypertension and hypothyroidism, and the primary gastric cancer treatment consisted of perioperative chemotherapy according to the fluorouracil plus leucovorin, oxaliplatin, and docetaxel (FLOT) protocol [2, 7]. According to a local oligometastatic treatment protocol, a left adrenalectomy was performed at the time of the gastrectomy due to a suspect adrenal metastasis detected on diagnostic imaging that had remained unchanged at preoperative evaluation imaging. The adrenal mass proved to be histologically benign with nodular hyperplasia, but no signs of malignant cells, while the pathology report of the gastrectomy specimen indicated high risk of recurrence, including a distressing ypTNM (pathological tumor–node–metastasis) stage after preoperative treatment, according to the American Joint Committee on Cancer (AJCC) staging manual 8th edition, and poor tumor regression according to Becker (Table 1) [8, 9].

**Table 1:** Pathology report of the gastrectomy specimen

	Description	Category	Details
Histology	Histological type	Adenocarcinoma	
	Lauren classification	Intestinal	
	Tumor location	Lower	
	Grade of differentiation	3	Poorly differentiated
	Lymphovascular invasion	LVI+	Positive
	Perineural invasion	PNI+	Positive
ypTNM	Primary tumor	ypT3	2 cm × 2 cm × 0.6 cm
	Regional lymph node involvement	ypN3a	Cancer in 13 out of 58 resected lymph nodes
	Distant metastatic spread	ypM0	No metastatic invasion of the left adrenal gland
	Extent of residual disease	R1	Tumor infiltration of the distal resection margin
TRG	Tumor regression grade	3	> 50% residual/vital tumor cells

Histological details, lymphovascular invasion (LVI), perineural invasion (PNI), pathological tumor-node-metastasis stage after preoperative treatment (ypTNM) classification according to the American Joint Committee on Cancer (AJCC) staging manual 8th edition, extent of residual disease (R), and tumor regression grade (TRG) according to Becker

**Fig. 1:**
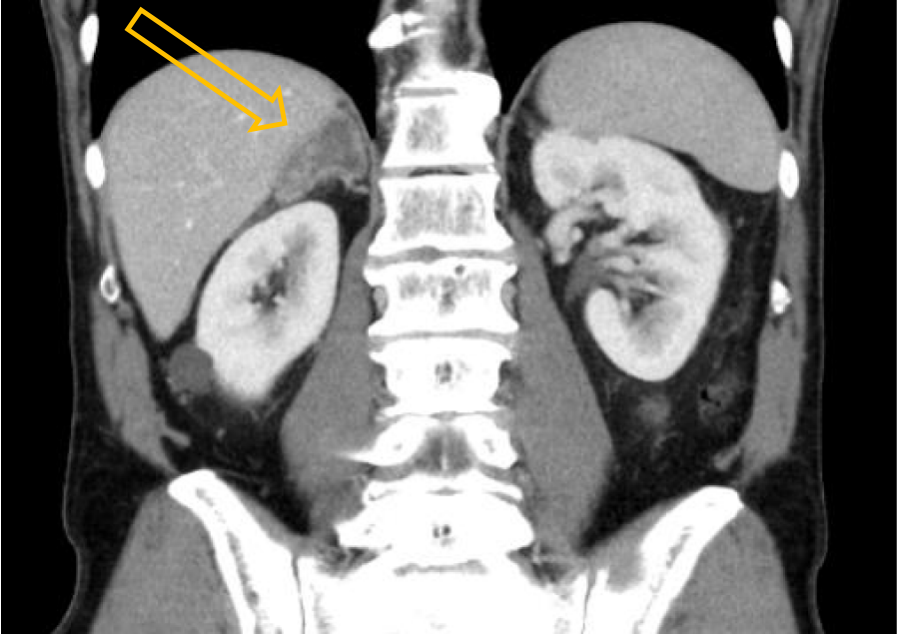
Routine 2-year control computed tomography (CT) of the upper abdomen. Consistent with a 3 cm × 6 cm right adrenal mass (arrow)

Physical examination revealed a tired, cachectic man reporting 8 kg of unintentional weight loss over the last months, skin hyperpigmentation, and low blood pressure (100/70 mmHg). Routine blood tests showed minor deviations of serum electrolytes; elevated plasma potassium (5.0 mmol/L, reference range 3.5–4.6 mmol/L) and decreased plasma sodium (135 mmol/L, reference range 137–145 mmol/L). A routine control computed tomography (CT) scan, performed 2 weeks prior to the visit, revealed a 3 cm × 6 cm right adrenal mass (Fig. 1).

The patient history of contralateral adrenalectomy, together with clinical symptoms and signs, biochemical findings, and imaging, was indicative of adrenal insufficiency. Primary adrenal insufficiency was confirmed by decreased serum cortisol levels (73 nmol/L at 9 am, reference range 135–540 nmol/L), increased plasma adrenocorticotropic hormone (ACTH) levels (89 pmol/L, reference range 1.6–14 pmol/L), and a positive (that is, no steroid response) ACTH stimulation test. Glucocorticoid replacement therapy (10 mg of hydrocortisone twice daily) was initiated in consultation with an endocrinologist, with rapid improvement in fatigue, normalized body weight, and normalized serum electrolytes. Pathological examination of an ultrasound-directed biopsy of the right adrenal gland was consistent with metastasis from the previously treated gastric adenocarcinoma. The cancer cell proliferation rate was high and the predictive biomarkers human epidermal growth factor receptor 2 (HER2) and programmed death ligand 1 (PD-L1), were negative. The final diagnosis was thereby primary adrenal insufficiency caused by surgical removal of the left and gastric cancer metastatic invasion of the right adrenal gland. Mineralocorticoid replacement therapy was not initiated after balancing the potential side effects of mineralocorticoid  therapy (edema, muscle weakness, electrolyte deviations) against the expected complications of advanced cancer and chemotherapy, together with the poor prognosis that metastasized gastric cancer entails.

**Fig. 2:**
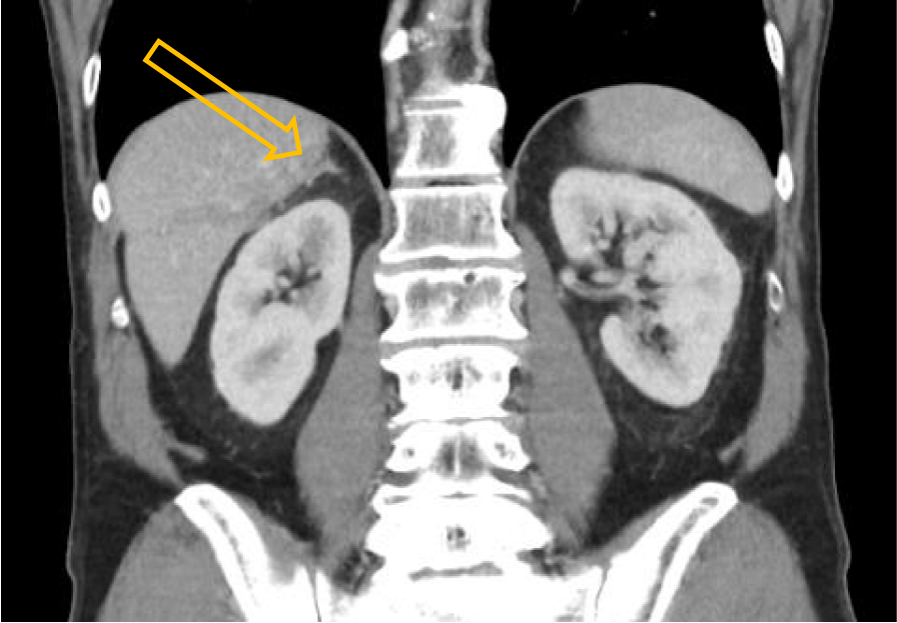
Therapy evaluation computed tomography (CT) of upper abdomen. With no radiological signs of extra-adrenal malignant disease and radiological response (shrinkage) of the right adrenal mass (arrow) after 3 months of first line palliative chemotherapy, followed by stereotactic body radiation therapy (SBRT) of the right adrenal gland

Radiological evaluation after 3 months (six cycles) of first line palliative chemotherapy, according to fluorouracil plus lecovorin, and irinotecan (FOLFIRI), showed no signs of extra-adrenal metastases and stable disease regarding the adrenal mass [10]. The latter was hence treated locally with stereotactic body radiotherapy (SBRT); 50 Gray in five fractions. The patient has thereafter been evaluated clinically together with radiology (CT, chest and abdomen) and laboratory tests (including a complete blood count, basic metabolic panel, liver panel, and the gastrointestinal cancer markers CEA, CA 19-9, and CA125) every 2–3 months. More than 17 months after the pathology-confirmed relapse of gastric adenocarcinoma, the patient is fully recovered and clinically well on glucocorticoid replacement therapy and with no radiological or biochemical signs of active malignant disease, except the residual right adrenal gland metastasis (Fig. 2).

## Discussion and conclusions

In this report, we present a case of adrenal insufficiency caused by surgical removal of the left adrenal gland in conjunction with distal gastrectomy due locally advanced gastric adenocarcinoma, followed by recurrent metastatic invasion of the right adrenal gland 2 years after primary treatment. The patient recovered fully on glucocorticoid replacement therapy and the metastatic disease was controlled during an unexpectedly long time period using a less conventional multimodal treatment approach: irinotecan-based doublet chemotherapy followed by stereotactic body radiotherapy (Additional file 1: Fig. S1).

Inherent weaknesses of case reports are the lack of reproducibility and generalizability. There is also a risk of overinterpreting the benefits of the described multimodal treatment approach in this frail patient population. We still believe that this case report merits publication since adrenal insufficiency induced by malignant disease and its treatment is probably not rare, and there is a risk of delayed clinical management since symptoms mimic those of advanced cancer. The reported long-term survival is indeed encouraging, and even if only a small subset of patients gain from this approach, it is worth reporting since large randomized controlled trials are highly unlikely to take place.

Primary adrenal insufficiency is, by definition, caused by innate dysfunction of the adrenal glands [5, 6]. One well-functioning adrenal gland is generally enough to support adequate hormonal secretion [11]. Common symptoms of adrenal insufficiency include fatigue, weight loss, abdominal pain, nausea, and vomiting [5, 6]. Skin hyperpigmentation and hypotension are typical clinical signs, while biochemical findings include hyponatremia and hyperkalemia [5, 6]. Symptoms of adrenal insufficiency are typically unspecific, causing a delay in diagnosis and treatment initiation, but an adrenal crisis with hypovolemic shock can also be the first clinical presentation [5, 6]. In case of adrenal crisis, acute presentation is typically preceded by physiological stress, such as surgery, trauma, or infection [5, 6]. There are three main goals in the diagnosis of adrenal insufficiency; to confirm low cortisol secretion, to determine if the insufficiency is primary or central, and to establish the underlying cause. The treatment of adrenal insufficiency is glucocorticoid and, in selected cases, mineralocorticoid replacement therapy [5, 6]. An adrenal crisis requires intravenous glucocorticoids and immediate fluid resuscitation, including correction of electrolyte disturbances [5].

Consequent to an abundant blood supply, the adrenal glands are a common location for distant metastases, most frequently from malignant melanoma, lung, breast, renal, and gastrointestinal cancers [12]. The prevalence of adrenal insufficiency due to bilateral metastases has been reported to be low (3–8%) [13]. To our knowledge, there exist no systematic reports of primary adrenal insufficiency, following unilateral adrenal metastasis, in patients with a history of contralateral adrenalectomy, as described herein. Historically, locoregional therapy, including of adrenal metastases, is rarely considered in the metastasized setting [14]. Surgical management, that is adrenalectomy, has, however, recently been shown to improve outcomes in selected patient populations [15]. SBRT, including CT‐based planning and image guidance to deliver high doses of radiation with high precision to target lesions, constitutes a less aggressive locoregional treatment alternative [16]. SBRT of adrenal metastases has been reported to be clinically meaningful when systemic disease is under control [17].

In summary, this case highlights the importance of recognizing the clinical and biochemical symptoms and signs of adrenal insufficiency in all health care facilities managing patients with cancer, but also the role of locoregional treatment in the oligometastatic setting. The first can prove complicated since the classic symptom picture (fatigue, weight loss, abdominal pain, nausea, and vomiting) of adrenal insufficiency overlaps with that of advanced, especially gastric, cancer [5, 6, 18]. Still, adrenal glands are common sites of metastatic invasion and, although rare, adrenal insufficiency is a potentially life-threatening condition that is crucial to recognize, diagnose, and treat, accurately and timely. Metastasized gastric adenocarcinoma carries a dismal prognosis, but multimodal treatment, including locoregional therapy of oligometastatic disease, can prove beneficial in selected cases with systemic therapy disease control.

## Supplementary Information

**Additional file 1: Figure S1.** Time line according to the 2013 CARE Checklist.

## Data Availability

Data sharing is not applicable since no datasets were generated or analyzed. No additional data or material is publicly available due to patient confidentiality.

## Abbreviations

AJCC: American Joint Committee on Cancer
CT: Computed tomography
FLOT: Fluorouracil plus leucovorin, oxaliplatin, and docetaxel
FOLFIRI: Fluorouracil plus leucovorin, and irinotecan
HER2: Human epidermal growth factor receptor 2
LVI: Lymphovascular invasion
PD-L1: Programmed death ligand 1
PNI: Perineural invasion
SBRT: Stereotactic body radiotherapy
TRG: Tumor regression grade
ypTNM: Pathological tumor–node–metastasis stage after preoperative treatment
