# Supplementary figures and images for: Adrenal insufficiency after curative-intent gastric cancer treatment: a case report
Source: J Med Case Rep. 2023 Apr 11;17:132. doi: 10.1186/s13256-023-03858-5 (PMC10088220; doi:10.1186/s13256-023-03858-5)

**Figure S1.** Time line according to the 2013 CARE Checklist.

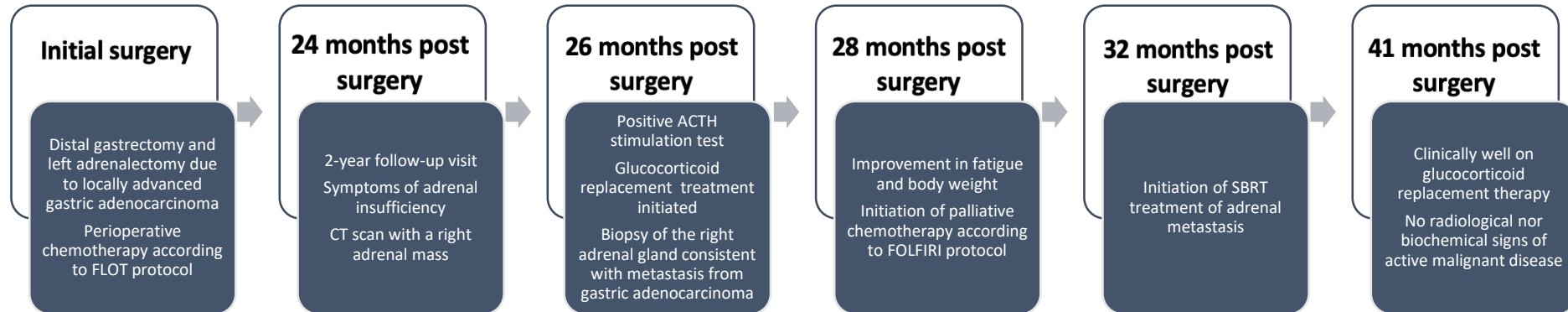

Supplement: Supplementary file 1 — Additional file 1: Figure S1. Time line according to the 2013 CARE Checklist. [file 13256_2023_3858_MOESM1_ESM.pdf]
